# Redox Biomarker Alterations and Disrupted Uric Acid–Catalase Activity Association in Gestational Diabetes Mellitus

**DOI:** 10.3390/antiox15070833

**Published:** 2026-07-01

**Authors:** Katarzyna Gawlik, Dorota Pawlica-Gosiewska, Tomasz Milewicz, Krystyna Słowińska-Solnica, Justyna Brodowicz, Dominik Żurek, Bogdan Solnica

**Affiliations:** 1 Department of Clinical Biochemistry, Jagiellonian University Medical College, Skawinska 8, 31-066 Krakow, Poland; dorota.pawlica@uj.edu.pl (D.P.-G.); krystyna.slowinska-solnica@uj.edu.pl (K.S.-S.); justyna.brodowicz@uj.edu.pl (J.B.); bogdan.solnica@uj.edu.pl (B.S.); 2 Department of Gynecological Endocrinology, Jagiellonian University Medical College, 31-501 Krakow, Poland; tomasz.milewicz@uj.edu.pl; 3 Department of Computer Science, Faculty of Computer Science, AGH University, 30-055 Krakow, Poland

**Keywords:** gestational diabetes mellitus, oxidative stress, redox balance, biomarkers, correlation analysis, antioxidant defense, uric acid, catalase

## Abstract

Gestational diabetes mellitus (GDM) is characterized by metabolic disturbances in which oxidative stress may play an important role. Most existing studies have examined individual biomarkers rather than their interrelationships. The present study evaluated selected oxidative stress, metabolic, and antioxidant markers, as well as their associations, in women with GDM compared with healthy pregnant controls. A total of 160 pregnant women (87 with GDM and 73 controls) were included. Biomarkers were measured, and their associations were assessed using correlation and interaction analyses. Women with GDM demonstrated higher levels of malondialdehyde (*p* < 0.001), leptin (*p* = 0.007), and ferric reducing antioxidant power (*p* < 0.001). The difference in the correlation between catalase activity and uric acid remained statistically significant after false discovery rate correction (*q* = 0.006), with a negative association in women with GDM and a positive association in controls. Interaction analysis further supported a group-dependent relationship between uric acid and catalase activity (*p* = 0.007; FDR-adjusted *q* = 0.007). These findings suggest that GDM may be associated not only with changes in individual biomarkers but also with alterations in selected redox-related relationships, indicating differences in redox regulation in GDM.

## 1. Introduction

Gestational diabetes mellitus (GDM) remains one of the most common metabolic complications of pregnancy. Defined by glucose intolerance with onset or first recognition during gestation [1], GDM is associated with long-term consequences for both mother and offspring, including an increased risk of type 2 diabetes and cardiometabolic disorders [1]. Despite its clinical relevance, the mechanisms underlying GDM development remain poorly understood.

Increasing evidence suggests that oxidative stress contributes to the pathophysiology of GDM [2]. Hyperglycemia promotes the overproduction of reactive oxygen species (ROS), leading to oxidative damage to various biomolecules [3].

Previous studies have reported alterations in biomarkers of oxidative damage to lipids, proteins, and DNA in women with GDM, including MDA, AOPP, and 8-OHdG [4,5,6,7,8]. Changes in adipokine concentrations, such as leptin and adiponectin [6,9], as well as alterations in antioxidant-related parameters, such as FRAP and catalase activity, have also been reported [6,10]. However, these biomarkers have most often been evaluated individually or in selected groups, and less is known about their interrelationships in GDM.

To address this gap, the present study evaluated a broader panel of biomarkers assessing complementary aspects of oxidative stress, metabolic regulation, and antioxidant status within the same cohort. In addition to comparing biomarker levels between women with GDM and healthy pregnant controls, the relationships between these parameters were assessed to determine whether GDM is associated with altered redox-related biomarker relationships. This approach may provide a more integrated view of redox alterations associated with GDM.

## 2. Materials and Methods

### 2.1. Study Population

The study included a total of 160 pregnant women, comprising 87 patients diagnosed with gestational diabetes mellitus and 73 healthy pregnant controls. All participants were consecutively recruited during routine or follow-up antenatal visits and examined in the third trimester of pregnancy (28–33 weeks of gestation).

Patients with GDM were recruited from the Diabetes Outpatient Clinic for Pregnant Women at the University Hospital in Krakow, Poland. The control group consisted of healthy pregnant women with normal glucose tolerance, recruited from a private obstetric practice in the same geographical area.

The diagnosis of GDM was established using a 75-g oral glucose tolerance test (OGTT) performed between 24 and 28 weeks of gestation, in accordance with the International Association of Diabetes and Pregnancy Study Groups (IADPSG) criteria [11]. GDM was diagnosed if at least one of the following plasma glucose thresholds was met or exceeded: fasting glucose ≥ 92 mg/dL (5.1 mmol/L), 1-h glucose ≥ 180 mg/dL (10.0 mmol/L), or 2-h glucose ≥ 153 mg/dL (8.5 mmol/L).

The following exclusion criteria were applied to both groups: pre-existing diabetes mellitus (type 1 or type 2), chronic or pregnancy-induced hypertension (including preeclampsia), multiple pregnancy, active infection, chronic inflammatory or autoimmune diseases, and chronic kidney or liver disorders.

The study was approved by the Bioethics Committee of the Jagiellonian University (No. 122.6120.259.2020). All participants provided written informed consent prior to enrollment, in accordance with the Declaration of Helsinki.

### 2.2. Clinical and Laboratory Assessments

Venous blood samples were collected after an overnight fast into serum tubes and K_2_-EDTA tubes. After centrifugation, serum and plasma were separated and stored at −80 °C until analysis.

Pre-pregnancy body weight and height were self-reported at enrollment. Maternal body weight during pregnancy was measured at the study visit using calibrated clinical scales. Body mass index (BMI) was calculated based on pre-pregnancy weight. Gestational weight gain was calculated as the difference between pre-pregnancy weight and body weight at the time of blood sampling.

Fasting serum glucose, uric acid, and total cholesterol concentrations were measured using enzymatic colorimetric methods with commercially available kits (Erba Mannheim, Mannheim, Germany) on an automated biochemical analyzer (Erba XL-180; Erba Mannheim, Mannheim, Germany).

Serum concentrations of leptin and adiponectin were determined using commercially available enzyme-linked immunosorbent assay (ELISA) kits (LDN Labor Diagnostika Nord GmbH & Co. KG, Nordhorn, Germany; and Mediagnost GmbH, Reutlingen, Germany, respectively).

Plasma levels of advanced oxidation protein products (AOPP), 8-hydroxy-2′-deoxyguanosine (8-OHdG), malondialdehyde (MDA), and catalase concentration were measured using ELISA kits (Bioassay Technology Laboratory, Shanghai, China; MyBioSource, San Diego, CA, USA; and Wuhan Fine Biotech Co., Ltd. (FineTest), Wuhan, China, respectively). All assays were performed according to the manufacturer’ instructions using a microplate reader (BioTek ELx808; BioTek Instruments, Winooski, VT, USA). MDA measurements were performed in a subset of participants due to limited sample availability.

The ferric reducing antioxidant power (FRAP) assay was performed according to the method described by Benzie and Strain [12], with minor modifications. For the FRAP assay, glacial acetic acid (>99.5%; Fluka, Buchs, Switzerland), anhydrous sodium acetate (POCH, Gliwice, Poland), TPTZ (Sigma-Aldrich, St. Louis, MO, USA), anhydrous ferric chloride (FeCl_3_; Sigma-Aldrich, St. Louis, MO, USA), hydrochloric acid (HCl, 36–38%; POCH, Gliwice, Poland), and ferrous sulfate heptahydrate (FeSO_4_·7H_2_O; Warchem Sp. z o.o., Zakręt, Poland) were used. The FRAP working reagent was freshly prepared by mixing acetate buffer (300 mM, pH 3.6), 2,4,6-tripyridyl-s-triazine (TPTZ) solution (10 mM in 40 mM HCl), and ferric chloride (FeCl_3_) solution (20 mM) in a 10:1:1 (*v*/*v*/*v*) ratio. The reagent was prewarmed to 37 °C prior to use and protected from light. Calibration curves were constructed using freshly prepared FeSO_4_·7H_2_O solutions in 10 mM HCl at concentrations of 0, 0.1, 0.2, 0.4, 0.8, and 1.6 mM. The assay was performed in a 96-well microplate. Briefly, 200 µL of FRAP reagent was added to each well, followed by 20 µL of plasma sample or standard solution. The mixture was incubated at 37 °C for 3 min. Absorbance was measured at 575 nm using a microplate reader (BioTek ELx808, BioTek Instruments, Winooski, VT, USA). A reagent blank (0 mM standard) was included. Results were calculated from the standard curve and expressed as mmol Fe^2+^ equivalents per liter of plasma.

Catalase activity in plasma was determined using a molybdate-based endpoint assay measuring residual hydrogen peroxide (H_2_O_2_), as described by Goth [13], with minor modifications. For the catalase activity assay, hydrogen peroxide (H_2_O_2_, 30%; POCH, Gliwice, Poland) and ammonium molybdate tetrahydrate ((NH_4_)_6_Mo_7_O_24_·4H_2_O; Warchem Sp. z o.o., Zakręt, Poland) were used. Briefly, 5 µL of plasma sample was mixed with 50 µL of H_2_O_2_ solution (30 mM) prepared in 50 mM phosphate buffer (pH 7.0). The reaction mixture was incubated at room temperature for 5 min. The reaction was terminated by adding 200 µL of ammonium molybdate solution (32.4 mM), forming a stable yellow complex with residual H_2_O_2_. Absorbance was measured at 405 nm using a microplate reader (BioTek ELx808, BioTek Instruments, Winooski, VT, USA). Catalase activity was calculated as the difference in absorbance between the control reaction (H_2_O_2_ without sample) and the tested sample, and expressed in units per milliliter (U/mL). One unit (U) of catalase activity was defined as the amount of enzyme decomposing 1 µmol of H_2_O_2_ per minute under the assay conditions. Under the assay conditions used (50 µL of 30 mM H_2_O_2_, 5 µL of sample, and 5 min incubation), catalase activity was calculated using the following equation: CAT (U/mL) = 60 × (ΔA/A_control).

### 2.3. Statistical Analysis

The normality of data distribution was assessed using the Shapiro–Wilk test. In both groups (GDM and control), only total cholesterol concentration and body height were normally distributed (*p* > 0.05), whereas the remaining variables were not (*p* < 0.05).

Accordingly, nonparametric tests were used for between-group comparisons. Differences between groups were evaluated using the Mann–Whitney U test. Effect size (r) was calculated as r = Z/√N. To account for multiple comparisons, the false discovery rate (FDR) was controlled using the Benjamini–Hochberg procedure. Correlations between variables were assessed using Spearman’s rank correlation coefficient (ρ).

For biomarkers with values that reached or exceeded the assay’s upper limit, the upper limit value was assigned and retained in the analyses. No observations were excluded solely because they reached or exceeded the upper limit. The number and percentage of observations that reached or exceeded the assay-specific upper limit were reported. These variables were additionally analyzed categorically by upper-limit status. Pearson’s chi-square test was used as the primary categorical comparison, while Fisher’s exact test was performed as a sensitivity analysis. For variables affected by ceiling effects, continuous analyses were interpreted with caution.

Differences between correlation coefficients obtained in independent groups (control vs. GDM) were evaluated using Fisher’s z transformation.

To further investigate relationships between variables (interaction analysis), multiple linear regression models including interaction terms (biomarker × GDM) were applied. Models were adjusted for pre-pregnancy BMI and fasting glucose concentration. These variables were selected as clinically relevant metabolic covariates potentially associated with redox-related biomarker levels and the investigated relationships. The number of covariates was limited to reduce the risk of overfitting and maintain model stability. Regression analyses were performed using raw, non-transformed biomarker values; therefore, regression coefficients (*β*) represent unstandardized estimates expressed in the original units. For interaction models, *q*-values were calculated for the two interaction terms within each adjustment set and reported alongside nominal *p*-values.

Sensitivity analyses were performed using two complementary approaches to evaluate the robustness of the interaction findings. All sensitivity models were based on the primary interaction models adjusted for pre-pregnancy BMI and fasting glucose concentration. First, to assess the influence of additional clinical and adipokine-related covariates, the primary OLS interaction models were further adjusted for maternal age and adiponectin concentration. Second, to assess the influence of data transformation and assay-related censoring within the BMI- and glucose-adjusted framework, log10-transformed models and, where applicable, Tobit regression models accounting for right-censoring at the upper limit of the assay measurement range were applied. For interaction analyses involving AOPP, additional sensitivity models were fitted after excluding observations reaching the AOPP upper limit. For log-transformed 8-OHdG models, log10(8-OHdG + 1) was used because three observations had a value of 0.

A nominal *p*-value < 0.05 was considered statistically significant before FDR correction. FDR correction was applied separately within each family of analyses: clinical and metabolic characteristics, biomarker comparisons, upper-limit categorical comparisons, correlation-difference analyses, and interaction analyses. After correction for multiple comparisons, *q* < 0.05 was considered statistically significant.

Statistical analyses were performed using R software (version 4.5.3; R Foundation for Statistical Computing, Vienna, Austria) in RStudio (version 2026.01.2+418; Posit Software, PBC) and Python (version 3.13.5; Python Software Foundation). Python-based analyses and selected figures were prepared using pandas 2.2.3, NumPy 2.3.5, SciPy 1.17.0, statsmodels 0.14.6, and Matplotlib 3.10.8.

## 3. Results

### 3.1. Clinical, Obstetric, and Metabolic Characteristics

Clinical, obstetric, and metabolic characteristics of the study groups are presented in Table 1. The groups were largely comparable across clinical, obstetric, and metabolic parameters; however, total cholesterol concentration was higher in women with GDM (Table 1). This difference remained significant after FDR correction.

In a subgroup analysis restricted to women with GDM and available treatment data (*n* = 74), no significant differences were observed between diet- and insulin-treated women in clinical, metabolic, adipokine, oxidative stress, or antioxidant parameters.

### 3.2. Adipokines, Oxidative Stress, and Antioxidant Biomarkers

Adipokines, markers of oxidative stress, and antioxidant status are presented in Table 2. Women with GDM exhibited higher levels of MDA, FRAP, and leptin, whereas no significant differences were observed for the remaining biomarkers. After FDR correction, differences in leptin, MDA, and FRAP remained statistically significant (Table 2).

The proportion of participants with values that reached or exceeded the upper limit of the assay measurement range did not differ significantly between groups after FDR correction. Catalase concentration showed a nominally significant between-group difference before FDR correction, with a higher proportion of values reaching or exceeding the upper limit in women with GDM than in controls; however, this difference did not remain statistically significant after FDR correction. No between-group differences were observed for AOPP or 8-OHdG (Table 3). Sensitivity analysis using Fisher’s exact test is presented in Supplementary Table S1.

### 3.3. Correlation Analysis

**Figure 1:**
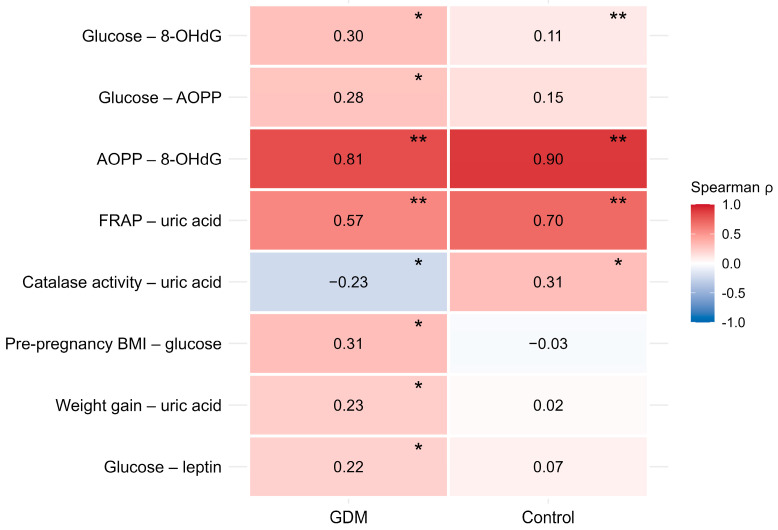
Heatmap of Spearman correlations between selected metabolic, oxidative stress, and antioxidant parameters in women with GDM and control groups. Spearman correlation coefficients (*ρ*) are presented within the cells. Color intensity represents the strength and direction of the correlation (blue: negative; red: positive). Statistically significant correlations based on unadjusted *p*-values are indicated as follows: * *p* < 0.05, ** *p* < 0.01. Between-group differences in correlation coefficients are reported in Supplementary Table S2 and visualized in Supplementary Figure S2. AOPP, advanced oxidation protein products; FDR, false discovery rate; GDM, gestational diabetes mellitus; 8-OHdG, 8-hydroxy-2′-deoxyguanosine.

Spearman correlation analysis revealed several associations between metabolic, oxidative stress, and antioxidant parameters (Figure 1; Supplementary Table S2).

In the GDM group, glucose was positively associated with oxidative stress markers, whereas these relationships were not statistically significant in the control group. Strong positive associations between AOPP and 8-OHdG were observed in both groups; however, this relationship did not differ significantly between groups (Table S2).

Among antioxidant-related parameters, FRAP was positively associated with uric acid in both groups and, in the GDM group, with total cholesterol; these associations did not differ significantly between groups (Table S2). Catalase activity showed opposite associations with uric acid in the two groups, being negative in GDM and positive in controls (*p* for difference < 0.001; Table S2). In the GDM group, catalase concentration was associated with its activity; no significant difference between groups was observed for this relationship (Table S2).

Pre-pregnancy BMI was positively associated with fasting glucose concentration in the GDM group but not in the control group. The between-group difference was nominally significant but did not remain significant after FDR correction (Table S2).

Overall, most correlations were consistent between groups; however, selected associations showed nominal between-group differences. After applying the Benjamini–Hochberg false discovery rate (FDR) correction for multiple comparisons, only the between-group difference in the correlation between catalase activity and uric acid remained statistically significant (Table S2; Figure S2).

### 3.4. Interaction Analysis

**Figure 2:**
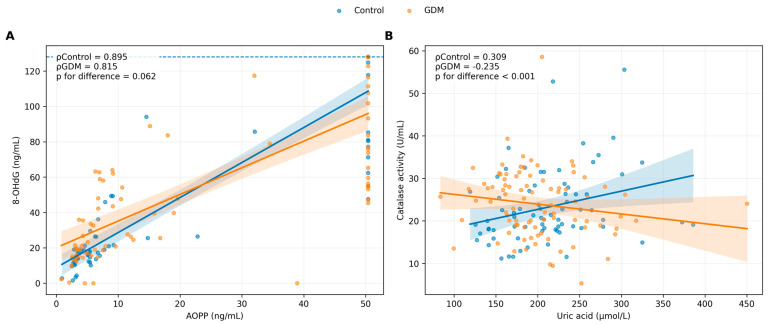
Interaction effects between selected biomarkers in women with GDM and controls. (**A**) Association between AOPP and 8-OHdG; (**B**) association between uric acid and catalase activity. Points represent individual observations. Lines represent fitted linear regression models for each group, and shaded areas indicate 95% confidence intervals. In panel (**A**), dashed lines indicate the upper limits of the assay measurement ranges for AOPP and 8-OHdG. Spearman correlation coefficients (*ρ*) and unadjusted *p*-values for between-group differences in the correlation coefficients are shown within each panel. AOPP, advanced oxidation protein products; GDM, gestational diabetes mellitus; 8-OHdG, 8-hydroxy-2′-deoxyguanosine.

In the primary models, a significant AOPP × GDM interaction was observed for 8-OHdG (Table 4; Figure 2A). Additionally, a significant uric acid × GDM interaction was observed for catalase activity (Table 4; Figure 2B). Both interactions remained statistically significant after FDR correction. After further adjustment for maternal age and adiponectin concentration, both interaction terms remained statistically significant: AOPP × GDM for 8-OHdG and uric acid × GDM for catalase activity (Table 4).

Sensitivity analyses indicated that the robustness of the interaction effects varied across biomarker pairs. The AOPP × GDM interaction for 8-OHdG remained significant in the Tobit model accounting for right-censoring, but did not remain statistically significant after log10 transformation or after exclusion of observations reaching the AOPP upper limit. In contrast, the uric acid × GDM interaction for catalase activity remained significant after log10-transformation of the outcome, supporting this association as the more consistent interaction finding (Supplementary Table S3).

## 4. Discussion

The present study demonstrates that gestational diabetes is associated with alterations in markers of oxidative stress, adipokine levels, and antioxidant parameters, as well as changes in the relationships among these processes. Women with GDM exhibited higher levels of MDA, leptin, and FRAP, suggesting increased oxidative stress, an altered adipokine profile, and changes in antioxidant capacity, potentially reflecting disturbances in systemic redox balance, as previously described in GDM [2]. Importantly, these findings indicate that GDM is associated not only with changes in individual biomarkers but also with alterations in redox system relationships.

Among the analyzed biomarkers, MDA showed the largest effect size (r = 0.54), indicating a substantial increase in lipid peroxidation in the GDM group. Median MDA levels were approximately 26% higher in women with GDM than in controls, supporting enhanced oxidative stress and aligning with previous studies reporting increased lipid peroxidation in hyperglycemic pregnancies [4,14]. Elevated MDA levels may reflect increased oxidative degradation of polyunsaturated fatty acids resulting from enhanced production of reactive oxygen species under hyperglycemic conditions [15]. Hyperglycemic conditions associated with GDM may promote oxidative stress by increasing metabolic flux and mitochondrial dysfunction, thereby contributing to membrane lipid oxidation [16]. Altered lipid metabolism may further enhance susceptibility to lipid peroxidation [4,14,17].

Interestingly, plasma FRAP levels were significantly higher in women with GDM (1.56 vs. 1.40 mmol/L, *p* < 0.001, *q* = 0.003, r = 0.27), representing an approximately 11% increase, despite elevated oxidative stress markers. This apparent paradox highlights a known limitation of the FRAP assay, which reflects the total reducing capacity of plasma rather than specific, regulated antioxidant mechanisms [12].

FRAP values are largely influenced by circulating low-molecular-weight antioxidants, particularly uric acid [12,18]. However, as serum uric acid levels did not differ significantly between groups, this factor alone does not explain the observed increase in FRAP. The elevated FRAP may reflect a compensatory response to increased oxidative stress or broader metabolic alterations, as previously described in pregnancy-related conditions such as pre-eclampsia [19].

Lipid-related metabolic changes may also partially account for the elevated FRAP values. A weak positive correlation between FRAP and total cholesterol was statistically significant only in the GDM group (*ρ* = 0.246, *p* < 0.05), suggesting that lipid-related factors may contribute to total reducing capacity [20]. Given that total cholesterol levels were also higher in the GDM group, this association may partially explain the elevated FRAP values observed in these women. Circulating lipophilic antioxidants, such as vitamin E (α-tocopherol), are transported within lipoproteins, linking their concentrations to lipid metabolism [21]. Accordingly, increased FRAP in GDM may reflect a combination of compensatory responses and broader alterations in metabolic homeostasis, including changes in lipid-related circulating components, rather than improved antioxidant defense.

Correlation analysis revealed a strong positive association between AOPP and 8-OHdG in both groups, indicating a relationship between oxidative protein modification and oxidative DNA damage [2,7,8]. Although this association was numerically weaker in women with GDM than in controls, the between-group difference was no longer statistically significant after FDR correction. This suggests that the relationship between oxidative protein modification and oxidative DNA damage was largely preserved in GDM.

By contrast, associations involving catalase activity were weaker and less consistent, suggesting that enzymatic antioxidant defense may be regulated differently from non-enzymatic antioxidant components. Notably, the association between catalase activity and uric acid reversed direction between groups: negative in women with GDM and positive in controls, and remained significant after FDR correction (*p* for difference < 0.001; *q* = 0.006). This observation was supported by interaction analysis, which showed that the uric acid × GDM interaction for catalase activity remained consistent across the reported models. This finding is particularly noteworthy, as previous studies have reported positive associations between uric acid and antioxidant parameters, including catalase activity [20].

However, this interpretation should be approached with caution, as uric acid has a well-recognized dual role in redox biology: while it contributes to antioxidant capacity in extracellular fluids [12,22], under conditions of hyperglycemia and metabolic stress, it has been shown to exert pro-oxidant and pro-inflammatory effects [22,23]. Accordingly, the altered catalase–uric acid relationship in GDM may reflect changes in antioxidant regulation as well as coexisting processes involving enhanced oxidative stress, indicating a broader shift in redox regulation under hyperglycemic conditions [2,3,24,25].

The redox alterations observed in the present study may also be considered in the broader context of other forms of diabetes, particularly type 2 diabetes. Increased lipid peroxidation and altered antioxidant defense have been reported in type 2 diabetes, where chronic hyperglycemia, insulin resistance, and metabolic disturbances contribute to oxidative stress [26,27,28]. In this respect, the higher MDA levels observed in women with GDM are consistent with a pattern also described in type 2 diabetes [29,30]. The increased leptin levels observed in our study may also reflect adipokine dysregulation associated with insulin resistance and metabolic stress, as reported in type 2 diabetes [31,32]. Antioxidant-related parameters appear to be more variable across diabetic populations. In our previous study, FRAP and uric acid concentrations were significantly higher in patients with type 2 diabetes than in healthy controls, and these parameters were strongly correlated [18]. By contrast, lower FRAP values have also been reported in type 2 diabetes [33,34]. These observations indicate that FRAP should be interpreted cautiously, as higher values may partly reflect the contribution of circulating reducing compounds, particularly uric acid, rather than improved antioxidant protection [12,18].

Although GDM differs from chronic forms of diabetes because it develops transiently within the specific metabolic environment of pregnancy, the partial overlap in redox disturbances suggests that oxidative imbalance may represent an early metabolic alteration associated with the later risk of type 2 diabetes [35,36].

Importantly, pre-pregnancy BMI was positively associated with fasting glucose concentration only in the GDM group. Although the between-group difference was nominally significant, it did not remain statistically significant after FDR correction. As neither pre-pregnancy BMI nor gestational weight gain differed between groups, this exploratory observation suggests that maternal metabolic status may be related to glycemic regulation in GDM, but this requires confirmation in larger studies.

Interaction analyses further supported group-dependent differences in selected redox relationships. The uric acid × GDM interaction for catalase activity was the more consistent finding, whereas the AOPP × GDM interaction for 8-OHdG was less consistent across sensitivity analyses and may have been influenced by observations that reached or exceeded the AOPP upper detection limit. Taken together, the findings suggest that GDM is associated with altered coordination within the redox network rather than solely with isolated changes in individual markers [2,24,25]. These observations highlight that appropriate analytical approaches are essential when interpreting biomarker interactions, particularly in the presence of assay-related censoring, where standard transformations alone may be insufficient to account for data limitations.

Finally, the absence of significant differences between women managed with diet and those receiving insulin should be interpreted cautiously. Incomplete treatment data and the potential influence of treatment initiated before blood sampling may have attenuated differences in metabolic control and redox-related biomarkers.

## 5. Limitations

Several limitations should be considered. First, the cross-sectional design precludes causal inference and the evaluation of redox dynamics across different stages of pregnancy. Although the overall sample included 160 participants, the statistical power for subgroup analyses may have been limited. In addition, some biomarker values reached or exceeded the assay’s upper detection limit, resulting in right-censored data that may have affected variability and the interpretation of selected associations. Although additional sensitivity analyses were performed to assess the potential influence of these measurement constraints, their impact cannot be ruled out entirely. Detailed dietary and lifestyle data, including coffee intake, dietary patterns, physical activity, and perceived stress, were not systematically collected and therefore could not be included in the regression models. Finally, subgroup analyses by treatment modality were limited by incomplete treatment data, including missing information on treatment type and, where applicable, on the duration and timing of treatment prior to blood sampling.

## 6. Conclusions

GDM was associated with higher levels of MDA, leptin, and FRAP, indicating differences in oxidative stress, the adipokine profile, and antioxidant-related parameters. The most consistent finding was the group-dependent relationship between uric acid and catalase activity, supported by both correlation and interaction analyses. Taken together, these results suggest that GDM is characterized not only by differences in individual biomarker levels but also by changes in selected redox-related relationships.

## Figures and Tables

**Table 1:** Clinical, obstetric, and metabolic characteristics of the study groups.

Variable	GDM (*n* = 87)	Controls (*n* = 73)	*p* Value	*q* Value (FDR)	Effect Size (r)
Maternal age (years)	32 (29–34)	31 (28–34)	0.815	0.973	0.02
Current pregnancy number	1 (1–2)	2 (1–2)	0.973	0.973	<0.01
Gestational age at sampling (weeks)	31 (30–32)	31 (30–33)	0.151	0.310	0.11
Pre-pregnancy BMI (kg/m^2^)	23 (20–27)	23 (20–25)	0.948	0.973	0.01
Weight gain (kg)	12 (10–14)	11 (9–13)	0.160	0.310	0.11
Fasting glucose (mmol/L)	4.62 (4.39–4.91)	4.52 (4.29–4.85)	0.194	0.310	0.10
Total cholesterol (mmol/L)	7.34 (6.24–7.97)	6.29 (5.64–7.27)	<0.001	0.008 *	0.27
Uric acid (µmol/L)	191.61 (163.75–227.19)	207.47 (171.46–236.19)	0.188	0.310	0.10

Data are presented as median (IQR). Differences between groups were assessed using the Mann–Whitney U test. Effect size was calculated as r = Z/√N. *q*-values were calculated using the Benjamini–Hochberg false discovery rate (FDR) procedure across the eight comparisons. BMI, body mass index; FDR, false discovery rate; GDM, gestational diabetes mellitus; IQR, interquartile range. * *q* < 0.05 was considered statistically significant.

**Table 2:** Adipokines, oxidative stress, and antioxidant biomarkers in women with GDM and controls.

Variable	GDM (*n* = 87)	Controls (*n* = 73)	*p* Value	*q* Value (FDR)	Effect Size (r)
Adiponectin (ng/mL)	5.29 (3.64–7.32)	3.92 (2.72–7.69)	0.084	0.151	0.14
Leptin (ng/mL)	45.05 (29.33–74.37)	34.39 (23.78–54.38)	0.007	0.022 *	0.21
Leptin/adiponectin ratio	8.42 (4.50–16.31)	8.25 (4.39–13.61)	0.495	0.495	0.05
MDA (nmol/mL) ^‡^	23.20 (21.03–30.01)	18.35 (15.22–20.34)	<0.001	<0.001 *	0.54
AOPP (ng/mL) ^†^	9.16 (4.32–50.40)	6.69 (3.50–50.40)	0.344	0.387	0.07
8-OHdG (ng/mL) ^†^	43.49 (18.61–78.95)	21.26 (14.66–85.26)	0.213	0.319	0.10
Catalase concentration (pg/mL) ^†^	83.50 (62.35–432.59)	74.11 (62.86–85.67)	0.064	0.143	0.15
FRAP (mmol/L)	1.56 (1.41–1.75)	1.40 (1.26–1.62)	<0.001	0.003 *	0.27
Catalase activity (U/mL)	24.55 (18.53–28.71)	20.73 (18.06–27.08)	0.248	0.319	0.09

Data are presented as median (IQR). Differences between groups were assessed using the Mann–Whitney U test. Effect size was calculated as r = Z/√N. q-values were calculated using the Benjamini–Hochberg false discovery rate (FDR) procedure across the nine comparisons. * *q* < 0.05 was considered statistically significant. ^†^ For values reaching or exceeding the upper limit of the assay measurement range, the assay-specific upper-limit value was used in the primary analyses. No observations were excluded solely because they reached or exceeded the upper limit. Additional categorical analyses based on upper-limit status are presented in Table 3 and Supplementary Table S1. ^‡^ MDA measurements were available for a subset of participants only (GDM, *n* = 40; controls, *n* = 40). AOPP, advanced oxidation protein products; FDR, false discovery rate; FRAP, ferric reducing antioxidant power; GDM, gestational diabetes mellitus; IQR, interquartile range; MDA, malondialdehyde; 8-OHdG, 8-hydroxy-2′-deoxyguanosine.

**Table 3:** Proportion of subjects with biomarker concentrations reaching or exceeding the upper limit of the assay measurement range in GDM and controls.

Variable	GDM (*n* = 87) *n* (%)	Controls (*n* = 73) *n* (%)	*p* Value	*q* Value (FDR)
AOPP ≥ upper limit (≥50.4 ng/mL)	28 (32.18%)	24 (32.88%)	0.930	0.930
8-OHdG ≥ upper limit (≥128 ng/mL)	11 (12.64%)	14 (19.18%)	0.260	0.390
Catalase concentration ≥ upper limit (≥2098.438 pg/mL)	13 (14.94%)	3 (4.11%)	0.024	0.072

Data are presented as *n* (%). Group differences were assessed using Pearson’s chi-square test. *q*-values were calculated using the Benjamini–Hochberg false discovery rate (FDR) procedure across the three comparisons. Exact sensitivity analyses are presented in Supplementary Table S1. AOPP, advanced oxidation protein products; FDR, false discovery rate; GDM, gestational diabetes mellitus; 8-OHdG, 8-hydroxy-2′-deoxyguanosine.

**Table 4:** Interaction analysis of selected associations in women with GDM and controls.

Outcome	Interaction Term	Adjusted for	*β*	*p* Value	*q* Value (FDR)
8-OHdG	AOPP × GDM	Pre-pregnancy BMI, fasting glucose	−0.517	0.005	0.007 *
8-OHdG	AOPP × GDM	Pre-pregnancy BMI, fasting glucose, maternal age, and adiponectin	−0.517	0.005	0.009 *
Catalase activity	Uric acid × GDM	Pre-pregnancy BMI, fasting glucose	−0.063	0.007	0.007 *
Catalase activity	Uric acid × GDM	Pre-pregnancy BMI, fasting glucose, maternal age, and adiponectin	−0.062	0.009	0.009 *

*β*, unstandardized regression coefficient for the interaction term. Models included both main effects and the interaction term and were adjusted for the covariates listed in the “Adjusted for” column. q-values were calculated using the Benjamini–Hochberg FDR correction within each adjustment set. * *q* < 0.05. Censoring-sensitive and transformed-model analyses are presented in Supplementary Table S3. AOPP, advanced oxidation protein products; BMI, body mass index; FDR, false discovery rate; GDM, gestational diabetes mellitus; 8-OHdG, 8-hydroxy-2′-deoxyguanosine.

## Data Availability

The data presented in this study are available on request from the corresponding author due to ethical restrictions.

## Supplementary Materials

The following supporting information can be downloaded at: https://www.mdpi.com/article/10.3390/antiox15070833/s1, Table S1: Categorical analyses of biomarkers reaching or exceeding the assay upper detection limit; Table S2: Differences in Spearman correlations between selected metabolic, oxidative stress, and antioxidant parameters in women with GDM and controls; Table S3: Sensitivity analyses of biomarker interaction models in women with GDM and controls; Figure S1: Proportion of values reaching or exceeding the assay upper detection limit in women with GDM and controls; Figure S2: Between-group differences in Spearman correlations.

## Abbreviations

The following abbreviations are used in this manuscript:

AOPP	Advanced oxidation protein products
BMI	Body mass index
FRAP	Ferric reducing antioxidant power
GDM	Gestational diabetes mellitus
MDA	Malondialdehyde
OGTT	Oral glucose tolerance test
ROS	Reactive oxygen species
8-OHdG	8-hydroxy-2′-deoxyguanosine
