# Supplementary material for: Redox Biomarker Alterations and Disrupted Uric Acid–Catalase Activity Association in Gestational Diabetes Mellitus
Source: Antioxidants (Basel). 2026 Jul 1;15(7):833. doi: 10.3390/antiox15070833 (PMC13404354; doi:10.3390/antiox15070833)
Supplement: Supplementary file 1 [file antioxidants-15-00833-s001.zip › antioxidants-4319922-supplementary.pdf]

Supplementary Materials

Table S1. Categorical analyses of biomarkers reaching or exceeding the upper limit of the assay measurement range.

Biomarker	Upper limit	GDM at or above the upper limit, n (%)	Control at or above the upper limit, n (%)	χ^2 *p*/*q* (FDR)	Fisher *p*/*q* (FDR)
AOPP (ng/mL)	50.4	28 (32.2%)	24 (32.9%)	0.930/0.930	1.000/1.000
8-OHdG (ng/mL)	128	11 (12.6%)	14 (19.2%)	0.260/0.390	0.281/0.422
Catalase concentration (pg/mL)	2098.44	13 (14.9%)	3 (4.1%)	0.024/0.072	0.032/0.096

Group differences were assessed using Pearson's chi-square test as the primary categorical analysis, with Fisher's exact test used as a sensitivity analysis. q-values were calculated using the Benjamini–Hochberg FDR procedure across the three comparisons. AOPP, advanced oxidation protein products; FDR, false discovery rate; GDM, gestational diabetes mellitus; 8-OHdG, 8-hydroxy-2′-deoxyguanosine.

Table S2. Differences in Spearman correlations between selected metabolic, oxidative stress, and antioxidant parameters in women with GDM and controls.

**Category**	**Variable pair**	**ρ GDM**	**ρ Control**	**$\Delta\rho$ (bootstrap 95% CI)**	***p*/*q* for difference**
Glucose and oxidative stress	Glucose – 8-OHdG	0.300	0.108	0.193 (-0.102 to 0.476)	0.213/0.355
Glucose and oxidative stress	Glucose – AOPP	0.281	0.149	0.132 (-0.180 to 0.420)	0.392/0.490
Oxidative stress parameters	AOPP – 8-OHdG	0.815	0.895	-0.079 (-0.185 to 0.013)	0.062/0.207
Antioxidant parameters	FRAP – uric acid	0.568	0.697	-0.129 (-0.341 to 0.070)	0.179/0.355
Antioxidant parameters	FRAP – total cholesterol	0.246	0.200	0.046 (-0.271 to 0.351)	0.767/0.767
Antioxidant parameters	Catalase activity – uric acid	-0.235	0.309	-0.544 (-0.820 to -0.243)	< 0.001/0.006**
Antioxidant parameters	Catalase activity – catalase conc.	0.284	0.209	0.075 (-0.242 to 0.381)	0.621/0.690
Metabolic parameters	Pre-pregnancy BMI – glucose	0.308	-0.029	0.337 (0.035 to 0.622)	0.032/0.160
Metabolic parameters	Weight gain – uric acid	0.233	0.023	0.210 (-0.098 to 0.512)	0.186/0.355
Metabolic parameters	Glucose – leptin	0.216	0.066	0.150 (-0.168 to 0.492)	0.343/0.490

ρ, Spearman's correlation coefficient; $\Delta\rho$, difference in Spearman's correlation coefficients between the GDM and control groups (ρGDM − ρControl). Differences between independent correlation coefficients were evaluated using Fisher's z transformation. Bootstrap confidence intervals were calculated for $\Delta\rho$. q-values were calculated using the Benjamini–Hochberg false discovery rate (FDR) correction across the 10 correlation-difference tests. Values in the final column are presented as p value/q value. AOPP, advanced oxidation protein products; BMI, body mass index; CI, confidence interval; FDR, false discovery rate; FRAP, ferric reducing antioxidant power; GDM, gestational diabetes mellitus; 8-OHdG, 8-hydroxy-2′-deoxyguanosine. ** $q < 0.01$.

Table S3. Sensitivity analyses of biomarker interaction models in women with GDM and controls.

Outcome	Interaction term	Model/sensitivity analysis	n	Censored n	β	SE	*p* value
8-OHdG	AOPP × GDM	Primary OLS, raw values	160	NA	-0.517	0.181	0.005**
8-OHdG	AOPP × GDM	OLS after log10 transformation†	160	NA	-0.158	0.092	0.090
8-OHdG	AOPP × GDM	Tobit for right-censored outcome	160	25	-0.649	0.214	0.002**
8-OHdG	AOPP × GDM	OLS excluding AOPP upper-limit observations	108	NA	-0.858	0.651	0.190
8-OHdG	AOPP × GDM	Tobit excluding AOPP upper-limit observations	108	1	-0.858	0.638	0.178
Catalase activity	Uric acid × GDM	Primary OLS, raw values	160	NA	-0.063	0.023	0.007**
Catalase activity	Uric acid × GDM	OLS after log10 transformation of outcome	160	NA	-0.001	0.000	0.007**

All models included the main effects and the interaction term and were adjusted for pre-pregnancy BMI and fasting glucose concentration. β values are unstandardized coefficients for the interaction term. Tobit models accounted for right-censoring of the outcome at the assay upper detection limit. †For 8-OHdG log-transformed models, log10(8-OHdG + 1) was used because three observations had a value of 0. Additional covariate-adjusted OLS models including maternal age and adiponectin are presented in Table 4. AOPP, advanced oxidation protein products; BMI, body mass index; GDM, gestational diabetes mellitus; NA, not applicable; OLS, ordinary least squares; SE, standard error; 8-OHdG, 8-hydroxy-2′-deoxyguanosine. ***p* < 0.01.

Figure S1. Proportion of values reaching or exceeding the upper limit of the assay measurement range in women with GDM and controls.

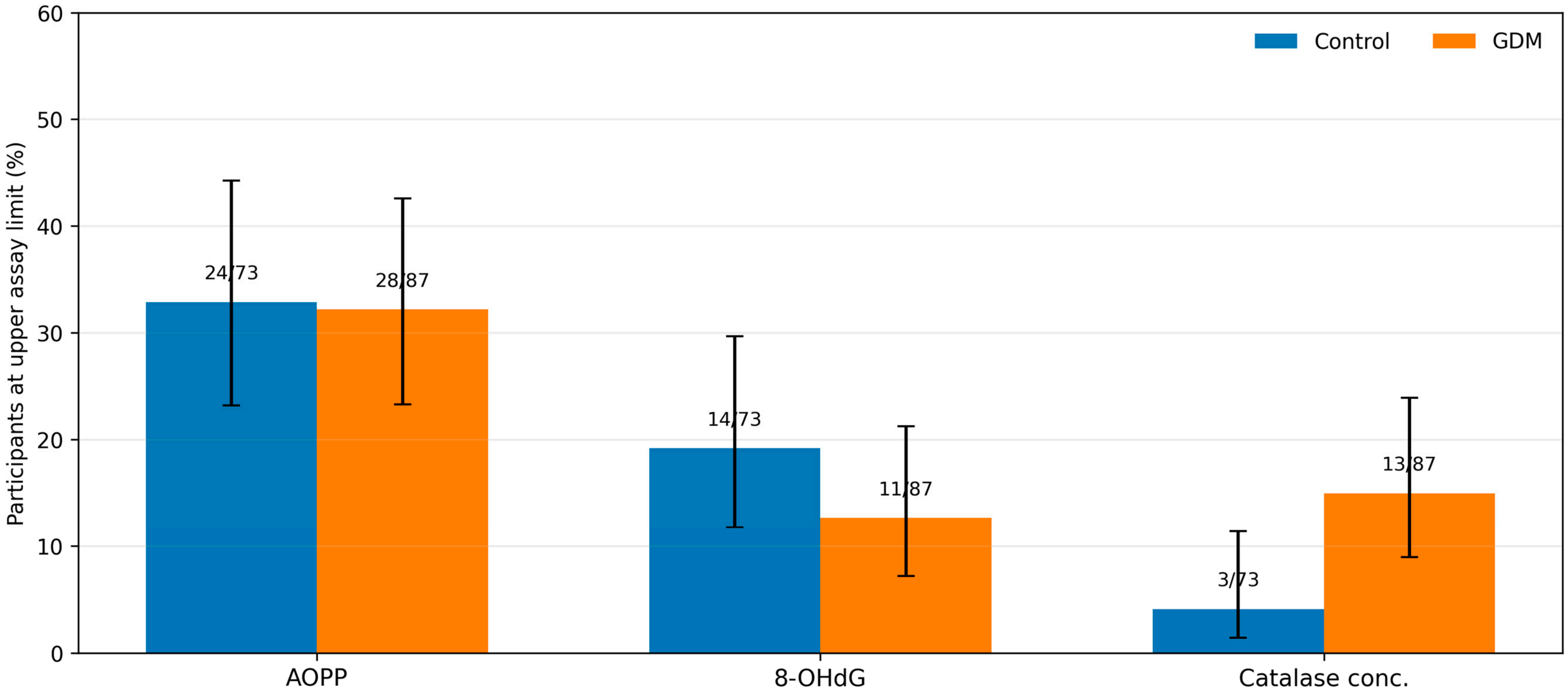

Bars represent the proportion of participants with values reaching or exceeding the upper limit of the assay measurement range. Error bars indicate Wilson 95% confidence intervals. Numbers above the bars indicate the number of observations reaching or exceeding the upper limit divided by the group-specific sample size. AOPP, advanced oxidation protein products; CI, confidence interval; GDM, gestational diabetes mellitus; 8-OHdG, 8-hydroxy-2′-deoxyguanosine.

Figure S2. Between-group differences in Spearman correlations.

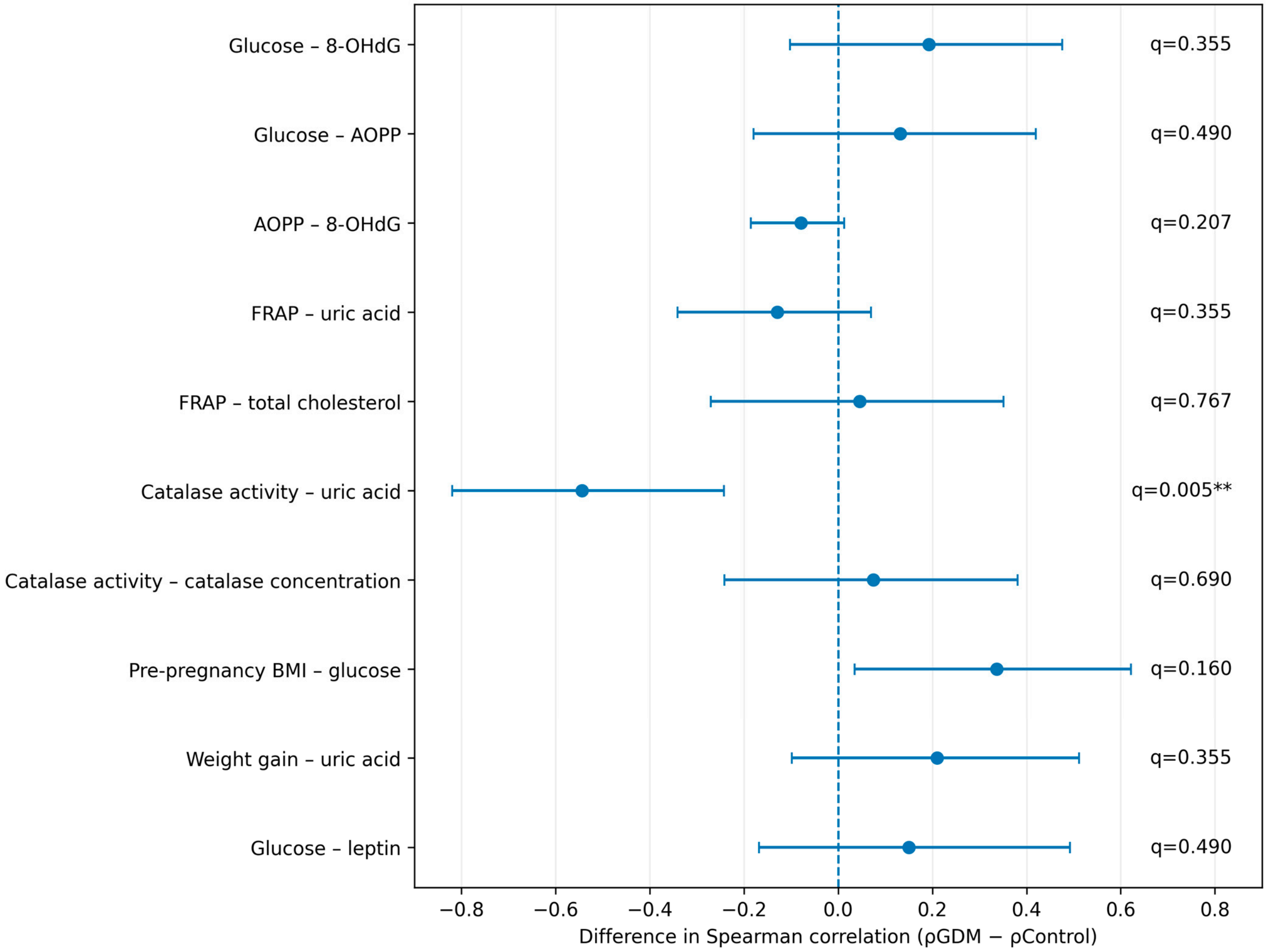

Points indicate Δϱ = ϱGDM − ϱControl; horizontal bars indicate bootstrap 95% confidence intervals. q-values are shown after Benjamini–Hochberg false discovery rate correction. Statistically significant differences after FDR correction are indicated as follows: ** $q < 0.01$. CI, confidence interval;

FDR, false discovery rate; GDM, gestational diabetes mellitus; ϱ, Spearman's correlation coefficient; Δϱ, difference in Spearman's correlation coefficients between the GDM and control groups.
